# Improving the Stability of Lithium Aluminum Germanium Phosphate with Lithium Metal by Interface Engineering

**DOI:** 10.3390/nano12111912

**Published:** 2022-06-03

**Authors:** Yue Zhang, Hanshuo Liu, Zhong Xie, Wei Qu, Jian Liu

**Affiliations:** 1 School of Engineering, Faculty of Applied Science, University of British Columbia, 3333 University Way, Kelowna, BC V1V 1V7, Canada; yue.zhang@ubc.ca; 2 Energy, Mining and Environment Research Center, National Research Council Canada, 4250 Wesbrook Mall, Vancouver, BC V6T 1W5, Canada; zhong.xie@nrc-cnrc.gc.ca (Z.X.); wei.qu@nrc-cnrc.gc.ca (W.Q.)

**Keywords:** lithium aluminum germanium phosphate, lithium metal anode, interface modification, atomic layer deposition

## Abstract

Lithium aluminum germanium phosphate (LAGP) solid electrolyte is receiving increasing attention due to its high ionic conductivity and low air sensitivity. However, the poor interface compatibility between lithium (Li) metal and LAGP remains the main challenge in developing all-solid-state lithium batteries (ASSLB) with a long cycle life. Herein, this work introduces a thin aluminum oxide (Al_2_O_3_) film on the surface of the LAGP pellet as a physical barrier to Li/LAGP interface by the atomic layer deposition technique. It is found that this layer induces the formation of stable solid electrolyte interphase, which significantly improves the structural and electrochemical stability of LAGP toward metallic Li. As a result, the optimized symmetrical cell exhibits a long lifetime of 360 h with an areal capacity of 0.2 mAh cm^−2^ and a current density of 0.2 mA cm^−2^. This strategy provides new insights into the stabilization of the solid electrolyte/Li interface to boost the development of ASSLB.

## 1. Introduction

Lithium metal is a promising anode candidate for constructing high-energy-density lithium batteries due to its high specific capacity (3860 mAh g^−1^) and low redox potential (−3.05 V vs. standard hydrogen electrode) [1,2]. Currently, its development is plagued by the intrinsic safety issues of liquid electrolytes because of the flammability of organic solvents used [3,4]. Replacing organic liquid electrolytes with non-flammable and highly conductive solid electrolytes (SEs) is an effective approach to improving battery safety [5,6]. With respect to this, there is growing research interest in the development of all-solid-state lithium-ion batteries (ASSLBs), and significant progress has been made over the past few years [7,8,9]. Critical characteristics of SEs include high lithium-ion conductivity, excellent chemical stability, and good mechanical properties [10]. Various SEs have been well developed, such as polymer-based SEs, garnet-type Li_7_La_3_Zr_2_O_12_ [11], sulfide [12], and NASICON-type LiM_2_(PO_4_)_3_ (M = Ti, Ge, Hf, Zr, Sn) [13], as well as composite SEs [14,15,16]. Aluminum (Al) -doped NASICON-type Li_1+x_Al_x_Ge_2-x_(PO_4_)_3_ (LAGP) oxides have received tremendous attention due to their high chemical and electrochemical stability in the air, high ionic conductivity (>10^−4^ S cm^−1^), and good mechanical strength [10]. The fast Li-ion conduction in the LAGP system benefits from the main Li-ion diffusion pathway along 36*f* and M_2_ interstitial sites resulting from Al^3+^ to Ge^4+^ substitution [17]. LAGP SEs have been synthesized by various routes, from melt-quenching [18], sintering [19], sol-gel [20], to hot-press [21], etc. Controlling key parameters such as crystallization and sintering temperature or pressure is critical in tailoring desirable highly-conductive LAGP pellets [21,22,23].

Li/LAGP interfacial incompatibility remains the main challenge for the further development of ASSLBs. Due to the high Fermi energy level of Li metal, germanium (Ge) in LAGP would be irreversibly degraded upon being contacted with Li metal at the Li/LAGP interface, resulting in an increased impedance versus time [24]. When an electrical current is applied to the Li metal anode, part of Li ions can be reduced at the LAGP side, leading to a local volume expansion which triggers cracks in the LAGP SE [25]. These cracks could cause the pulverization of SEs, mechanical deterioration, or even cell failure (short circuit) [26]. Consequently, the unstable Li/LAGP interface causes inferior cell capacity and lifetime, which need to be mitigated using effective interfacial stabilization strategies.

Li et al. [27] introduced a succinonitrile-based plastic interlayer between Li metal and LAGP by in situ solidification. This interlayer isolated the direct contact between Li and LAGP and regulated uniform Li-ion distribution, which enabled a 240-h cycle life in symmetrical Li cells. Zhou et al. [28] sputtered an amorphous Ge thin film on the LAGP surface, which ensured intimate Li/LAGP contact and suppressed the unexpected Ge reduction, thus contributing to a superior symmetrical cell over 100 cycles at 0.1 mA cm^−2^. Xiong et al. [29] created a quasi-solid-state paste interlayer composed of LAGP nanoparticles and ionic liquid, which enabled fast Li-ion conduction and improved chemical stability toward metallic Li, and suppressed thermal runaway. Sun et al. [30] adopted the atomic layer deposition (ALD) technique to coat ultrathin aluminum oxide (Al_2_O_3_) on the surface of Li_1.3_Al_0.3_Ti_1.7_(PO_4_)_3_ (LATP), and the Li/LATP interface was significantly stabilized at 0.01 mA cm^−2^. Currently, most Li/LAGP/Li symmetrical cells show a long cycle time at a low current density of 0.1 mA cm^−2^ [1,3,25,26,28,31,32,33,34,35,36,37], making it hard to meet the demand for practical applications. Improving the electrochemical stability of LAGP at higher current densities is an urgent task in the development of high-energy-density ASSLBs [38].

In this work, the effect of sintering temperature on the structure and ionic conductivity of LAGP is investigated, and 850 °C is found as the optimal sintering temperature to reach an ionic conductivity of 2.4 × 10^−4^ S cm^−1^ at room temperature. Furthermore, an ultrathin Al_2_O_3_ coating layer is deposited in the LAGP pellet surface via the ALD technique, which physically isolates the contact between Li metal and LAGP SE and enhances the electrochemical stability of LAGP toward metallic Li. The Li symmetrical cell with Al_2_O_3_-coated LAGP SE showed an excellent lifetime of 360 h at a current density of 0.2 mA cm^−2^ and capacity density of 0.2 mAh cm^−2^, which is higher than most previously reported Li/LAGP/Li cells with interlayer modification.

## 2. Materials and Methods

### 2.1. Materials

LAGP powders with a stoichiometric formula of Li_1.5_Al_0.5_Ge_1.5_(PO_4_)_3_ were purchased from the Shanghai Institute of Ceramics (Shanghai, China). The precursors for ALD were trimethylaluminium (TMA) and deionized water (H_2_O). Lithium hexafluorophosphate (LiPF_6_, Gotion, CAS: 21324-40-3), ethylene carbonate (EC, Gotion, CAS: 96-49-1), and diethyl carbonate (DEC, Gotion, CAS: 105-58-8) were used as received for coin cell assembly.

### 2.2. Preparation of LAGP Pellets

LAGP pellets were made by a conventional dry-pressing method (15T Compact Hydraulic Pellet Press, MTI Corporation, Richmond, CA, USA). To begin, 0.2 g LAGP of powder was placed into a stainless-steel die with 12 mm diameter and pressed at 200 MPa uniaxial pressure at room temperature for 2 min. The obtained pellets were then transferred into a ceramic crucible. The sintering process was conducted at 800–900 °C in the air for 6 h at a heating rate of 2° min^−1^ in a tube furnace. The thickness of the as-prepared LAGP pellets was about 0.8 mm.

### 2.3. Atomic Layer Deposition of Al_2_O_3_ on LAGP

The Al_2_O_3_ layer was coated on LAGP in a commercial ALD reactor (GEMStar™ XT Atomic Layer Deposition Systems, Singapore). The coating process was carried out at 100 °C by following a TMA pulse/TMA purge/H_2_O pulse/H_2_O purge sequence in each cycle. After 50 cycles, Al_2_O_3_ films were deposited on LAGP noted as LAGP@Al_2_O_3_50.

### 2.4. Materials Characterizations

The morphologies and microstructures of LAGP powder and pallets were observed and analyzed by scanning electron microscopy (SEM) and energy dispersive spectroscopy (EDS). X-ray diffraction (XRD) was used to analyze the crystal structure of LAGP. Surface elemental analysis was investigated by X-ray photoelectron spectroscopy (XPS).

Electrochemical impedance spectroscopy EIS measurements were carried out at 25 °C and elevated temperatures (30–60 °C). The LAGP pellets were Au-coated on both sides as electrodes and clamped for conduction. The ionic conductivity (*σ*) of LAGP was measured by Equation (1):

(1) $$\sigma =\frac{L}{RA}$$

where *L* and *A* are the thickness and effective area of LAGP, respectively, and *R* is the resistance obtained by EIS) testing within a frequency range of 0.01–10^6^ Hz with an AC amplitude of 5 mV.

### 2.5. Electrochemical Measurements

CR2032 cells were assembled in an argon-filled glovebox workstation with H_2_O and O_2_ less than 0.1 ppm. First, 10 µL electrolyte (1 M LiPF_6_ in EC: DEC) were added to each Li/LAGP/Li cell. Symmetrical Li/LAGP/Li cells were tested in NEWARE battery cycler (CT-4008T-5V50mA-164, Shenzhen, China) at room temperature and 40 °C with current densities ranging from 0.02 to 0.2 mA cm^−2^ with a plating-striping time of 1 h. The cycled Li/LAGP batteries were disassembled in the argon-filled glove box and then characterized by SEM and XPS to collect surface composition information.

## 3. Results and Discussion

**Figure 1:**
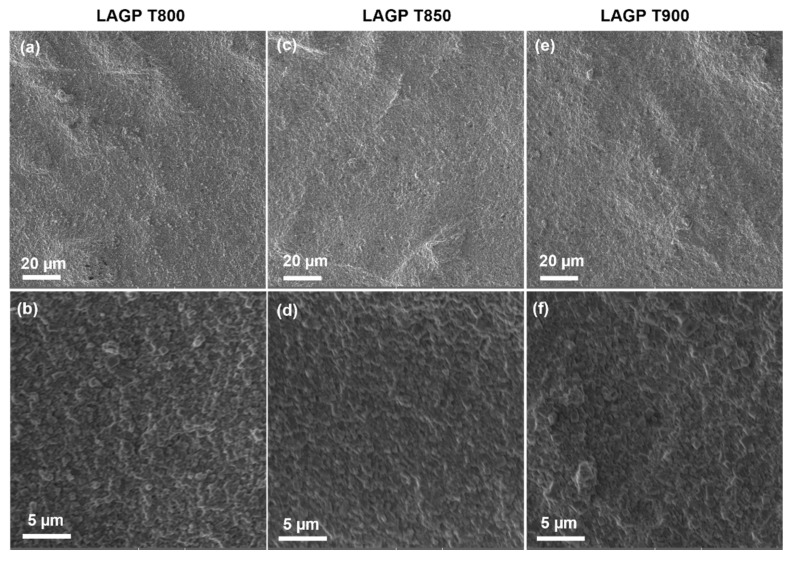
SEM images of LAGP pellets sintered at (**a**,**b**) 800 °C, (**c**,**d**) 850 °C, and (**e**,**f**) 900 °C.

Figure 1 presents the SEM images of LAGP pellets sintered at 800, 850, and 900 °C, respectively. Most LAGP particles have small sizes of less than 1 µm, as shown in Figure S1. After being pressed and sintered at high temperatures (800–900 °C), LAGP particles were closely stacked to form a dense surface, and no pores were found in higher-magnification images, ensuring continuous Li-ion pathways to achieve high ionic conductivity. The ionic conductivity of LAGP solid electrolyte relies on the Li-ion conduction in both bulk (σ_bulk_) and along the grain boundary (σ_gb_). With the sintering temperature increasing, unit cell volumes would grow to form wider Li-ion migration channels, thus leading to higher bulk conductivity σ_bulk_ [23,39]. The Li-ion conduction along the grain boundary (σ_gb_) mainly depends on grain-grain ceramics contact and density. High sintering temperatures above 850 °C would possibly induce cracks due to grain strain, decrease ceramics density, and slow down Li-ion transport at the grain boundary [23,40,41]. Consequently, LAGP sintered at 850 °C showed the highest ionic conductivity.

**Figure 2:**
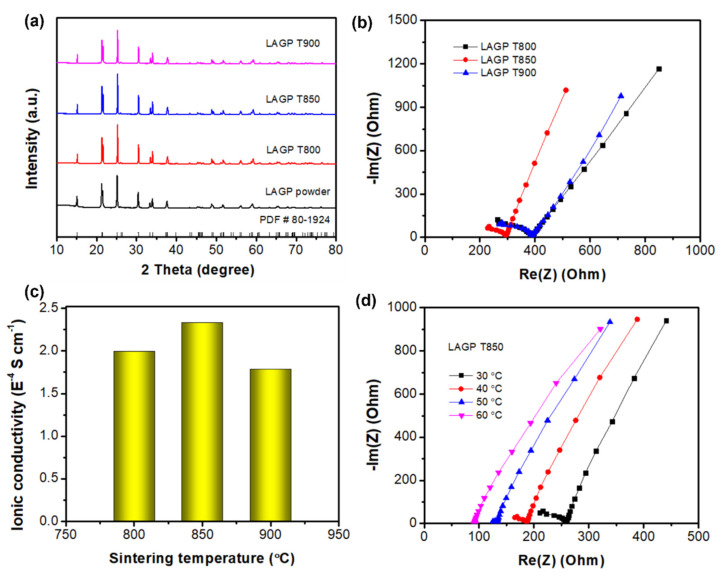
(**a**) XRD patterns, (**b**) Nyquist plots, (**c**) ionic conductivities of LAGP T800, LAGP T850, and LAGP T900 pellets, (**d**) Nyquist plots of LAGP T850 at 30–60 °C.

XRD patterns of LAGP pellets sintered at 800–900 °C are shown in Figure 2a. All the characteristic peaks of LAGP pellets match well with the LAGP powders and can be indexed as LiGe_2_(PO_4_)_3_ (JCPDS PDF No. 80-1924). Figure 2b gives the Nyquist plot of LAGP pellets sintered at 800–900 °C measured in a frequency range of 0.01–1 × 10^6^ Hz at room temperature. LAGP T850 possesses a smaller electrochemical impedance (283 Ω) than LAGP T800 (390 Ω) and T900 (392 Ω), thus leading to higher ionic conductivity of 2.4 × 10^−4^ S cm^−1^ (vs. 2.0 × 10^−4^ and 1.8 × 10^−4^ S cm^−1^ for T800 and T900, respectively) as illustrated in Figure 2c. Further, the relationship between impedance and temperature for LAGP T850 is plotted in Figure 2d. It could be found that the impedance markedly decreases from 260 to 92 Ω with temperature increasing from 30 to 60 °C, corresponding to the ionic conductivity from 2.6 × 10^−4^ to 7.3 × 10^−4^ S cm^−1^. LAGP T850 will be selected as a reference for further electrochemical stability tests with Li metal due to its highest ionic conductivity.

**Figure 3:**
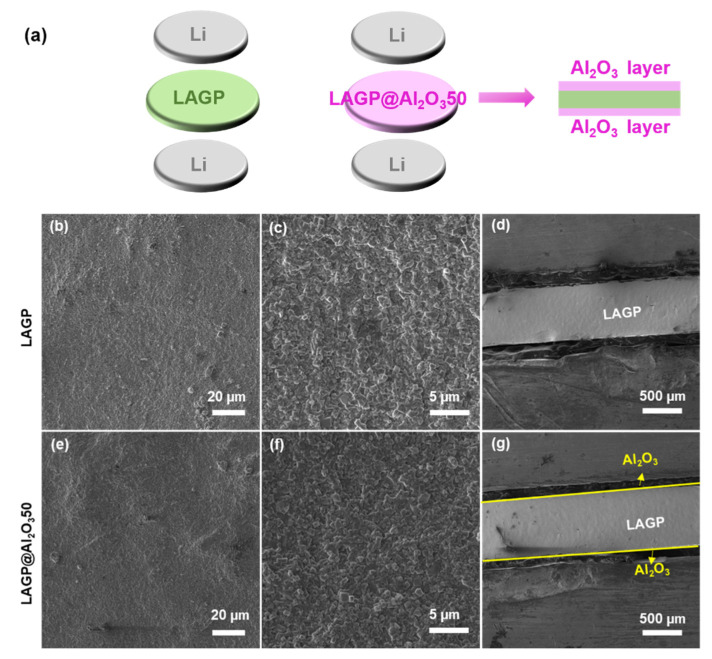
(**a**) Schematic illustration of Li/LAGP/Li and Li/LAGP@Al_2_O_3_/Li cell configurations, top-view and cross-sectional SEM images of (**b**–**d**) LAGP and (**e**–**g**) LAGP@Al_2_O_3_50.

Figure S2 shows a voltage-time profile of Li/LAGP/Li symmetrical cell at room temperature. It could be found that the voltage hysteresis is gradually increasing at a low current density of 0.02 mA cm^−2^ and rapidly rises to 3 V at only 0.04 mA cm^−2^. The large overpotential can be attributed to the unstable Li/LAGP interface and Li dendric deposition [26]. Herein, the Al_2_O_3_ layer is deposited on the surface of LAGP, as illustrated in Figure 3a, which can separate LAGP from the Li metal to prevent side reactions. The thickness of the Al_2_O_3_ layer is controlled at 5 nm based on our previous study on the P2-Na_0.66_(Mn_0.54_Co_0.13_Ni_0.13_)O_2_ cathode [42]. Figure 3b–g displays the SEM images of LAGP and LAGP@Al_2_O_3_50 from the top view and cross-section. Both demonstrate a compact and flat surface without pores. There is no noticeable change found in the surface and cross-section morphology.

**Figure 4:**
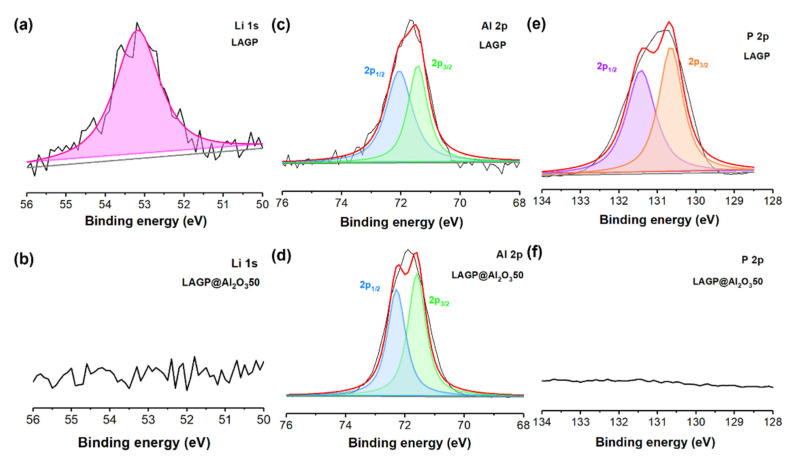
XPS spectra of (**a**,**b**) Li 1s, (**c**,**d**) Al 2p, and (**e**,**f**) P 2p of LAGP and LAGP@Al_2_O_3_50.

XPS characterization was carried out to investigate the surface chemistry of LAGP with and without the Al_2_O_3_ layer. As shown in Figure S3 and Table S1, Ge, P, and Li elements are only detected on bare LAGP surface and absent for LAGP@Al_2_O_3_50, suggesting the successful deposition of Al_2_O_3_ film on the LAGP surface. The prominent peak in Li 1s XPS spectra (Figure 4a) is assigned to Li oxide of LAGP [43], while it is not detectable in LAGP@Al_2_O_3_50 (Figure 4b). Al 2p peaks can be resolved into 2p_1/2_ and 2p_3/2_ peaks corresponding to the existence of Al in LAGP and ALD-Al_2_O_3_ (Figure 4c,d). Similarly, P is only observed in bare LAGP (Figure 4e) and is absent for Al_2_O_3_-coated LAGP (Figure 4f). Therefore, it can be concluded that the thin Al_2_O_3_ layer by ALD is successfully deposited on the surface of LAGP without significant morphology change. The Al_2_O_3_ layer is expected to induce a stable interface between Li and LAGP, suppress side reactions, and enhance the electrochemical stability of LAGP toward metallic Li.

**Figure 5:**
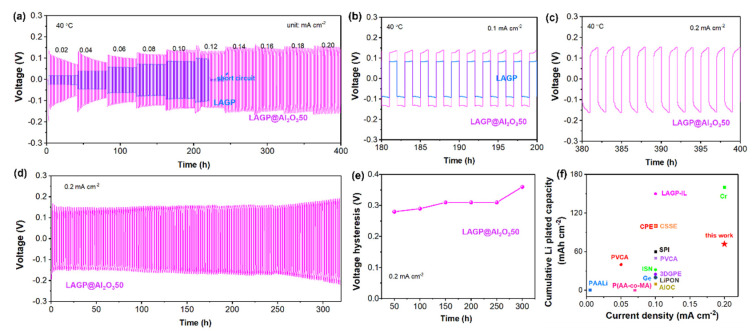
(**a**) Rate performance of symmetrical Li cells with bare and Al_2_O_3_-coated LAGPs as SEs at 0.02–0.2 mA cm^−2^ at 40 °C, voltage profiles at (**b**) 0.1 and (**c**) 0.2 mA cm^−2^, (**d**) long-term cycling performance of the Li/LAGP@Al_2_O_3_50/Li cell at 0.2 mA cm^−2^ (areal capacity: 0.2 mAh cm^−2^) and (**e**) corresponding voltage hysteresis, (**f**) comparison of cumulative Li plated capacity from this work and literatures.

Figure 5a shows voltage profiles of symmetrical Li cells with bare and Al_2_O_3_-coated LAGP SEs at 0.02–0.2 mA cm^−2^ at 40 °C. Li/LAGP/Li cell has a lower overpotential at the initial current density of 0.02 mA cm^−2^, which becomes larger with the increase in current density and finally fails at 0.12 mA cm^−2^. In comparison, the Li/LAGP@Al_2_O_3_50/Li cell exhibits a stable voltage hysteresis after initial cycles and undergoes a repeated Li plating/stripping process at a much higher current density of 0.2 mA cm^−2^. The steady voltage profiles of LAGP@Al_2_O_3_50 (Figure 5b,c) indicate the formation of a stable solid electrolyte interphase (SEI) between Li and LAGP. Long-term cycling performance is evaluated for the Li/LAGP@Al_2_O_3_50/Li cell, which exhibits a long lifetime of 320 h at the areal capacity of 0.2 mAh cm^−2^ and current density of 0.2 mA cm^−2^ (Figure 5d). The voltage hysteresis is mostly stabilized at 0.3 V until 250 h and then shows a slight increase, as illustrated in Figure 5e. It should be noted that the rate test is performed following the cycling test from the same Li/LAGP@Al_2_O_3_50/Li cell. Consequently, this cell has a 360-h Li plating/stripping duration at 0.2 mAh cm^−2^.

Moreover, Table 1 and Figure 5f compare cumulative Li plated capacity of previous Li/LAGP/Li cells and this work in the cycling test. It can be seen that most of the previous studies proposed polymer interlayer to modify Li/LAGP interface and assessed the cycling performance at 0.1 mA cm^−2^, which demonstrated smaller cumulative Li plated capacity below 60 mAh cm^−2^. Although the Li/Cr-LAGP-Cr/Li [3] cell showed a high Li plated capacity of 160 mAh cm^−2^, the overpotential is as large as 1.2 V upon Li plating/stripping behaviors. In comparison, this work employs an inorganic Al_2_O_3_ layer to stabilize Li/LAGP interface, and the cell possesses a much smaller overpotential of 0.3 V at 0.2 mA cm^−2^.

**Figure 6:**
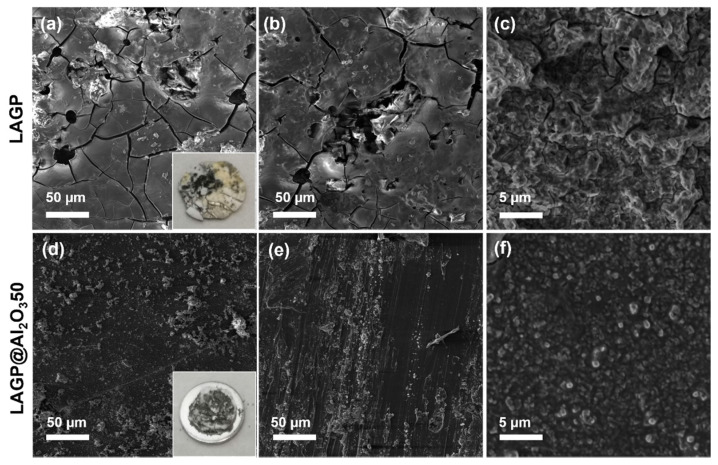
Top-view SEM images of cycled (**a**–**c**) LAGP and (**d**–**f**) LAGP@Al_2_O_3_50. The insets of (**a**,**d**) show the photos of cycled LAGP and LAGP@Al_2_O_3_50.

To reveal the internal structural change after repeated cycles, the cycled Li/LAGP/Li and Li/LAGP@Al_2_O_3_50/Li were disassembled for further analysis. Figure 6 shows the SEM images and digital photos of cycled LAGP and LAGP@Al_2_O_3_50. Abundant pores and cracks are observed in cycled-bare LAGP at different magnifications, and the cycled LAGP surface turns yellow, implying possible Ge^4+^ reduction after being in contact with Li metal [25]. The pores and cracks may originate from continuous Li dendrite deposition at the unstable Li/LAGP interface and ultimately cause a short circuit failure [25,26]. On the contrary, Al_2_O_3_-coated LAGP illustrates a flat and dense surface after cycling, indicating the generation of stable SEI. This Al_2_O_3_-induced SEI regulates Li dendrites and induces a reliable symmetrical Li cell. Therefore, the deposited thin Al_2_O_3_ film is beneficial for stabilizing the Li/LAGP interface and regulating Li dendrites.

**Figure 7:**
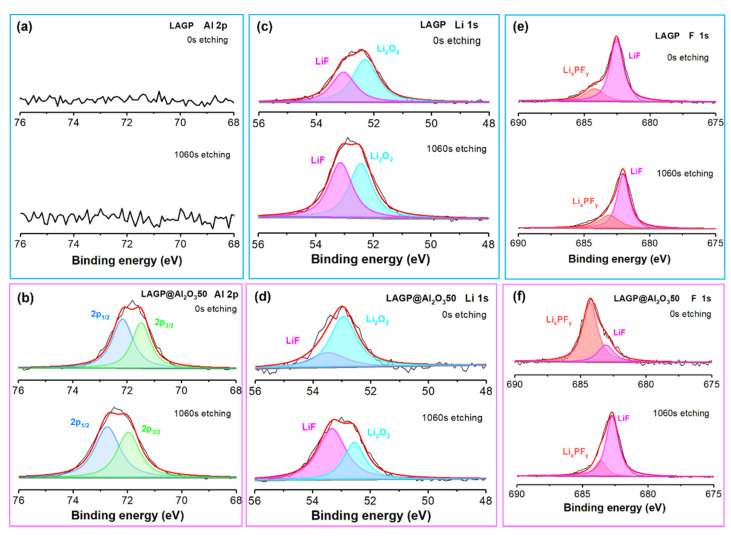
XPS spectra of (**a**,**b**) Al 2p, (**c**,**d**) Li 1s, (**e**,**f**) F 1s of the surface of LAGP and LAGP@Al_2_O_3_50 after cycling test.

Meanwhile, a XPS test was performed to collect surface elemental information of cycled LAGP and LAGP@Al_2_O_3_50. As shown in Figure S4 and Figure 7a, and Table S2, the Al signal is absent in bare LAGP, implying the formation of an Al-deficient SEI layer. Al 2p spectra in Figure 7b can be assigned to two peaks at 72.2 and 71.5 eV, respectively, which slightly shift to 72.7 and 72.0 eV with 1060 s etching (corresponding to about 8.8 nm depth from the top of the surface). Since the SEI layers are usually ~70 nm or thicker [46,47], both the 0 s and 1060 s etching spectra should come from the SEI layer instead of the LAGP SE. Meanwhile, the absence of Al signal from the cycled bare LAGP sample also indicates that the 1060 s etching does not reach the bulk LAGP SE. The peak shift of Al 2p spectra reveals the compositional variation in the SEI layer along the vertical direction of cycled LAGP@Al_2_O_3_50. The surface Li 1s spectrum (0 s etching) of cycled bare LAGP (Figure 7c) can be decomposed into two peaks located at 53.1 and 52.3 eV, respectively, corresponding to LiF and Li_2_O_2_ in the SEI layer. The surface Li 1s spectrum (0 s etching) obtained from LAGP@Al_2_O_3_50 shows higher binding energies of 53.5 and 53.0 eV for LiF and Li_2_O_2_ (Figure 7d). It should be noted that the Li 1s spectrum from cycled LAGP@Al_2_O_3_50 surface has a smaller LiF/Li_2_O_2_ ratio than cycled bare LAGP, suggesting the different SEI compositions from the coated and uncoated LAGPs. A higher LiF/Li_2_O_2_ ratio is observed in the Li 1s spectrum of cycled LAGP@Al_2_O_3_50 after 1060 s etching, implying a gradient distribution of LiF/Li_2_O_2_ in the SEI layer. F 1s spectra in Figure 7e,f originate from Li_x_PF_y_ and LiF, which could be ascribed to the decomposition of the small addition of liquid electrolyte (LiPF_6_ in EC:DEC) [37]. The LiF/Li_x_PF_y_ ratio becomes larger for the LAGP@Al_2_O_3_50 after 1060s etching, which also indicates the compositional variation in the SEI layer along with the depth. The XPS results suggest that the SEI layers formed on the bared LAGP and Al_2_O_3_ coated LAGP possess different compositions. The Al_2_O_3_ coating induces an Al-containing SEI layer [48,49], formed on the surface of LAGP after repeated Li plating/stripping cycles. This unique SEI layer enhances the electrochemical stability of LAGP SE and extends the lifetime of Li/LAGP/Li symmetrical cells.

## 4. Conclusions

In summary, a thin Al_2_O_3_ film was deposited on the surface of LAGP pellet by the ALD method to address the issue of Li/LAGP interface incompatibility. The optimized Li/LAGP@Al_2_O_3_50/Li cell maintained a stable voltage profile up to 0.2 mA cm^−2^ and exhibited a 360-h cycling duration at the capacity density of 0.2 mAh cm^−2^. Remarkably, this cell showed a superior cumulative Li plated capacity of 72 mAh cm^−2^ at 0.2 mA cm^−2^ current density. SEM and XPS characterizations suggest that Al_2_O_3_ film induced the formation of an Al-rich SEI layer to regulate Li dendrite deposition and contributes to a uniform and dense interface after long Li plating/stripping cycles.

## Figures and Tables

**Table 1:** Comparison of Li/LAGP/Li cells with interface modification.

Cell Configuration	Interface Modification Layer	Cycling Performance	Cumulative Li Plated Capacity (mAh cm^−2^)	References
Li/PP-LAGP-PP/Li *	PP	500s @0.25 mA	-	[31]
Li/SPI-LAGP-SPI/Li *	SPI, by in situ solidification, 10 µm	600 h @0.1 mA cm^−2^	60	[1]
PAALi/LAGP/PAALi *	PAALi + LAGP	100 h @0.005 mA cm^−2^	0.5	[32]
Li/P(AA-co-MA)-LAGP- P(AA-co-MA)/Li	P(AA-co-MA), by spray-coating, 1.5 µm	20,000 s @0.07 mA cm^−2^	0.39	[25]
Li/PVCA-LAGP-PVCA/Li *	LiTFSI/PVCA,	500 h @0.1 mA cm^−2^	50	[33]
Li/AIOC-LAGP-AIOC/Li	AIOC, by spin coating, 11 µm	100 h @0.1 mA cm^−2^	10	[34]
Li/CPE-LAGP-CPE/Li	CPE, by spin coating	1000 h @0.1 mA cm^−2^	100	[36]
Li/lSN-LAGP-lSN/Li *	SN + LLZAO + FEC + LiTFSI, by in situ solidification	320 h @0.1 mA cm^−2^	32	[27]
Li/Ge-LAGP-Ge/Li	Ge, by puttering, 60 nm	200 h @0.1 mA cm^−2^	20	[28]
Li/Cr-LAGP-Cr/Li	Cr, by sputtering, 30 nm	800 h @0.2 mA cm^−2^	160 (overpotential: 1.2 V)	[3]
Li/LAGP-IL-LAGP-LAGP-IL/Li	LAGP-IL	1500 h @0.1 mA cm^−2^	150	[29]
Li/3DGPE-LAGP-3DGPE/Li *	PVDF-HFP + PEGDE + DPPO, 100 µm	250 h @0.1 mA cm^−2^	25	[44]
Li/CSSE-LAGP-CSSE/Li	PVC + TPU + LiTFSI, 50 µm	1000 h @0.1 mA cm^−2^	100	[45]
Li/PVCA-LAGP-PVCA/Li	PVCA + FEC	800 h @0.05 mA cm^−2^	40	[37]
Li/LiPON-LAGP-LiPON/Li	LiPON, by sputtering, 3 µm	200 h @0.1 mA cm^−2^	20	[26]
Li/Al_2_O_3_-LAGP- Al_2_O_3_/Li *	Al_2_O_3_, by ALD, 5 nm	360 h @0.2 mA cm^−2^	72 (overpotential: 0.3 V)	This work

*: 10 µL liquid electrolyte was used, or the interlayer was immersed in liquid electrolyte.

## Data Availability

Data is contained within the article or supplementary material.

## Supplementary Materials

The following supporting information can be downloaded at: https://www.mdpi.com/article/10.3390/nano12111912/s1. Figure S1: SEM image of LAGP powder, Figure S2: Voltage-time profile of the Li/LAGP/Li cell tested at 25 °C, Figure S3: XPS survey spectra of LAGP and LAGP@Al_2_O_3_ before the cycling test, Figure S4: XPS survey spectra of LAGP and LAGP@Al_2_O_3_ after the cycling test, Table S1: Elemental composition of LAGP and LAGP@Al_2_O_3_50 from XPS before battery tests, Table S2: Elemental composition of LAGP and LAGP@Al_2_O_3_50 from XPS after cycling tests.
