# Supplementary material for: Improving the Stability of Lithium Aluminum Germanium Phosphate with Lithium Metal by Interface Engineering
Source: Nanomaterials (Basel). 2022 Jun 3;12(11):1912. doi: 10.3390/nano12111912 (PMC9182743; doi:10.3390/nano12111912)
Supplement: Supplementary file 1 [file nanomaterials-12-01912-s001.zip › nanomaterials-1738000-supplementary.pdf]

nanomaterials

Supporting information

Improving the Stability of Lithium Aluminum Germanium Phosphate with Lithium Metal by Interface Engineering

Yue Zhang [1], Hanshuo Liu [2,*], Zhong Xie [2], Wei Qu [2] and Jian Liu [1,*]

[1] School of Engineering, Faculty of Applied Science, University of British Columbia, 3333 University Way, Kelowna, BC V1V 1V7, Canada; yue.zhang@ubc.ca
[2] Energy, Mining and Environment Research Center, National Research Council Canada, 4250 Wesbrook Mall, Vancouver, BC V6T 1W5, Canada;
zhong.xie@nrc-cnrc.gc.ca (Z.X.); wei.qu@nrc-cnrc.gc.ca (W.Q.)
* Correspondence: hanshuo.liu@nrc-cnrc.gc.ca (H.L.); jian.liu@ubc.ca (J.L.)

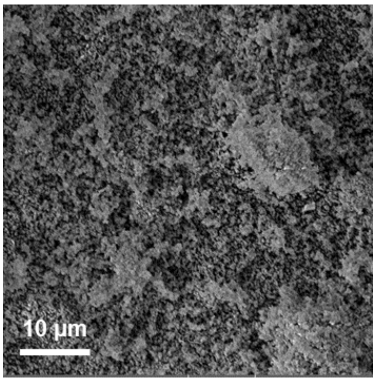

Figure S1. SEM image of LAGP powder.

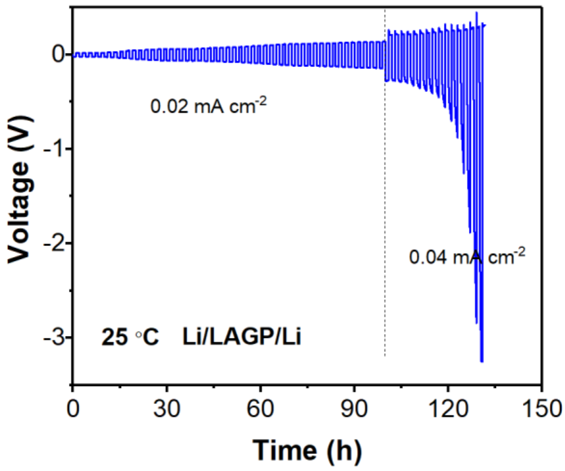

Figure S2. Voltage-time profile of the Li/LAGP/Li cell tested at 25 °C.

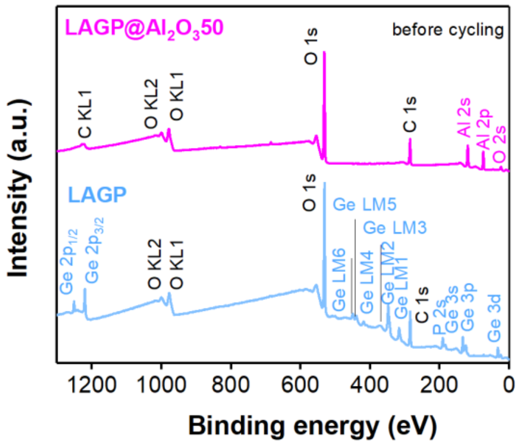

Figure S3. XPS survey spectra of LAGP and LAGP@Al_2O_3 before the cycling test.

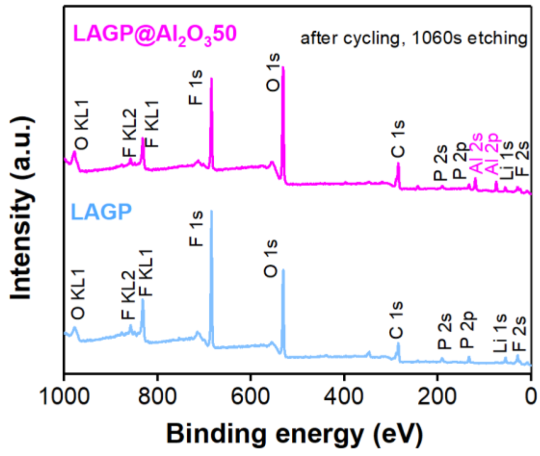

Figure S4. XPS survey spectra of LAGP and LAGP@Al_2O_3 after the cycling test.

Table S1. Elemental composition of LAGP and LAGP@$Al_2O_3$50 from XPS before battery tests.

	Weight percentage (wt%)									
	F	O	C	P	Ge	Si	Al	Ca	N	Li
LAGP	0.38	35.04	20.95	17.12	20.25	0.61	2.23	--	--	3.41
LAGP@Al_2O_3 50	0.9	39.41	19.07	--	--	0.3	39.83	0.24	0.26	--

Table S2. Elemental composition of LAGP and LAGP@$Al_2O_3$50 from XPS after cycling tests.

	Weight percentage (wt%)								
	F	O	N	Ca	C	S	P	Li	Al
LAGP	16.47	24.3	0.45	2.05	47.11	0.92	2.1	6.59	-
LAGP (1060s etching)	34.31	25.78	0.36	2.36	17.46	0.67	4.64	14.43	-
LAGP@$Al_2O_3$50	7.98	36.45	0.55	0	34.86	--	1.54	6.89	11.73
LAGP@$Al_2O_3$50 (1060s etching)	19.71	30.24	1.01	0.61	16.96	--	7.15	10.93	13.39
